# Myelodysplastic syndrome with neutrophilic panniculitis: A report of two cases and a literature review

**DOI:** 10.3892/ol.2015.2932

**Published:** 2015-02-04

**Authors:** LIREN QIAN, JIANLIANG SHEN, JIAN CEN, WENJIE YIN, YUANYUAN MA

**Affiliations:** Department of Hematology, Navy General Hospital, Beijing 100048, P.R. China

**Keywords:** myelodysplastic syndrome, neutrophilic panniculitis, neutrophilic dermatoses, corticosteroid

## Abstract

Myelodysplastic syndrome (MDS) associated with neutrophilic panniculitis (NP) is rare. To the best of our knowledge, there are only five previously reported cases of MDS associated with NP, consisting of four adults and a young male. The present study reviews the literature of the previously reported cases and presents two case reports of adult male patients who were initially diagnosed as MDS. The two patients presented with a sudden onset of fever without infection and erythematous indurated papules. The patients were diagnosed with NP by skin biopsy and their symptoms disappeared completely following treatment with systemic corticosteroids. NP may be an independent high-risk factor for MDS. However, due to the rarity of such cases, the exact pathogenesis of NP associated with MDS requires further investigation.

## Introduction

Myelodysplastic syndrome (MDS) is a disease entity encompassing a spectrum of clonal myeloid disorders characterized by ineffective hematopoiesis, cytopenias, qualitative disorders of blood cells and their precursors, clonal chromosomal abnormalities and a variable predilection to undergo clonal evolution to florid acute myeloid leukemia (AML) (1). MDS may be associated with a wide spectrum of neutrophilic dermatoses (NDs), including Sweet’s syndrome (SS), pyoderma gangrenosum (PG), subcorneal pustular dermatoses (Sneddon-Wilkinson disease) and erythema elevatum diutinum (EED), which may be classified by the predominant localization of the neutrophilic infiltrate (Table I) (1,2). Neutrophilic panniculitis (NP) is another condition that has been previously reported in five cases (1–5), including four adults and a young male. The present study presents two case reports of patients with MDS associated with NP and reviews the literature on this rare condition. Written informed consent was obtained from both patients.

## Case reports

### Patient one

A 35-year-old Chinese man presented to the outpatient clinic of the Navy General Hospital (Beijing, China) with a 5-month history of recurrent high fever. Based on bone marrow morphology, the patient was diagnosed with MDS of the French-American-British (FAB) subtype refractory cytopenia with multilineage dysplasia (RCMD) and displayed widespread erythematous nodules on the extremities and waist. The physical examination revealed numerous painful, erythematous nodules randomly distributed over the extremities and waist. No mucosal involvement was present. The nodules appeared along with the recurrence of high fever.

Further evaluation revealed hepatomegaly and splenomegaly without palpable adenopathy. The laboratory examinations revealed macrocytic anemia [hemoglobin (Hb), 8.3 g/dl; mean corpuscular volume (MCV), 106.6 fl], polymorphonuclear leukocytosis [white blood cell (WBC) count, 1.8×10^9^/l], a platelet count of 3×10^9^/l and a C-reactive protein level of 164.0 mg/l (normal, <8 mg/l). The erythrocyte sedimentation rate (ESR) was elevated to ≤72 mm/h. The ferritin levels were increased to >1,500.0 μg/l. Cryoglobulin, antinuclear antibodies, rheumatoid factor and antineutrophilic cytoplasmic antibodies (ANCA) were all negative. The C3 and C4 complement levels were normal. A chest computed tomography (CT) scan revealed clear lung fields. Immunofluorescence was negative for immunoglobulin and complement deposition. Repeated cultures for bacteria, fungi and tuberculosis of the pustular lesions were all negative. The blood and urine cultures were also negative. A biopsy of an erythematous nodule on the leg of the patient revealed the presence of NP.

The intermittent high fever and erythematous skin nodules were resistant to systemic antibiotics (meropenem plus teicoplanin) administered for 2 weeks. Corticosteroid therapy was initiated with methylprednisolone (1 mg/kg) based on the diagnosis of NP associated with MDS. Treatment led to the rapid improvement of the skin symptoms and fever. Following gradual steroid treatment discontinuation, the widespread skin lesions disappeared and did not recur. However, intermittent fever persisted and methylprednisolone was reintroduced. A bone marrow biopsy was performed 1 year later and revealed no evidence of progression to AML. The patient survived for 16 months and remained alive at the time of the present study.

### Patient two

A 46-year-old Chinese man presented to the outpatient clinic of our hospital with a 1-month history of recurrent high fever. Based on bone marrow morphology, the patient was diagnosed with MDS of the FAB subtype RCMD and displayed widespread erythematous nodules on the lower limbs and feet. The physical examination revealed several painful erythematous nodules randomly distributed over the lower limbs and feet. No mucosal involvement was present. The nodules appeared along with the recurrence of high fever.

Further evaluation revealed the absence of hepatomegaly, splenomegaly, or palpable adenopathy. The laboratory examinations revealed macrocytic anemia (Hb, 6.9 g/dl; MCV, 108.3 fl), polymorphonuclear leukocytosis (WBC, 1.1×10^9^/l), a platelet count of 7×10^9^/l and a C-reactive protein level of 122.6 mg/l (normal, <8 mg/l). The ESR was elevated to ≤48 mm/h. The ferritin levels were normal. Cryoglobulin, antinuclear antibodies, rheumatoid factor and ANCA were all negative. The C3 and C4 complement levels were normal. A chest CT scan revealed clear lung fields. Immunofluorescence was negative for immunoglobulin and complement deposition. Repeated cultures for bacteria, fungi and tuberculosis of the pustular lesions were all negative. The blood and urine cultures were also negative. A biopsy of an erythematous nodule on the foot of the patient revealed the presence of NP. The intermittent high fever and erythematous skin nodules were resistant to systemic antibiotics (ceftazidime) administered for 1 week. Corticosteroid therapy was initiated with methylprednisolone (1 mg/kg) based on the diagnosis of NP associated with MDS. Treatment led to the rapid improvement of the skin symptoms and fever. Following gradual steroid treatment discontinuation, the widespread skin lesions disappeared and did not recur. However, intermittent fever persisted and methylprednisolone was reintroduced. A bone marrow biopsy was performed 3 years later and revealed no evidence of progression to AML. The patient survived for 48 months and remained alive at the time of the present study.

## Discussion

MDS is a heterogenous group of stem cell disorders commonly characterized by ineffective hematopoiesis, cytopenias, qualitative disorders of blood cells and their precursors, clonal chromosomal abnormalities and a variable predilection to undergo clonal evolution to florid AML (1,2,6). MDS may be associated with a wide spectrum of skin lesions, including SS, PG, subcorneal pustular dermatoses and EED (7–10); however, it is rarely associated with NP, although this condition has been previously described in the literature.

NP is characterized by a lobular infiltrate of neutrophils and the absence of morphological abnormalities and belongs to the group of NDs, which are inflammatory skin diseases characterized by a cutaneous infiltrate of normal polymorphonuclear leukocytes without a known cause (2). NP may precede and even suggest the diagnosis of MDS, which may appear during follow-up. In certain cases, the appearance of cutaneous manifestations in patients with MDS may be the first sign of disease progression to AML. Skin involvement in MDS is of prognostic and therapeutic significance in adults (6). However, whether there is an association between NP and MDS remains controversial (1).

To the best of our knowledge, there are only five previously reported cases of MDS associated with NP, including four adults and a young male (1–5). Although Sutra-Loubet *et al* (2) reported an association of NP with MDS in 6 out of 8 patients, NP first appeared in addition to atypical SS and atypical PG in a study published by Matsumura *et al* (3), which described a 74 year-old patient with MDS and NP, whose panniculitis was responsive to steroid therapy. Chen *et al* (1) also reported the case of a 59-year-old patient with MDS and NP. The NP disappeared completely following treatment with systemic corticosteroids. However, the patient succumbed to septic shock complicated by lung infection 16 months later. Sutra-Loubet *et al* (2) reported the case of a patient with NP associated with myelodysplasia, whose NP disappeared following treatment with systemic and topical corticosteroids. Hendrickx *et al* (4) reported the case of a 13-year-old male patient with panniculitis of the right foot as the presenting sign for the ultimate diagnosis of MDS-AML, who was successfully treated with allogeneic bone marrow transplantation. Becherer *et al* (5) described the case of a 66-year-old patient with MDS with abnormal nuclear forms who developed NP; they suggested that NP development may be explained by the increased granulocyte colony-stimulating factor levels.

The pathogenesis of NP associated with MDS remains unknown. NP may precede and even suggest the diagnosis of MDS, which may appear during follow-up (4,5). A previous study suggested that abnormal neutrophil chemotactic activity may be an underlying mechanism (5), but the present cases did not receive such treatment during hospitalization.

The NP of the two patients reported in this study was successfully treated with systemic steroids (Table II). However, the symptoms of NP were recurrent and MDS persisted. In the abovementioned study by Hendrickx *et al* (4), the panniculitis post-bone marrow transplantation was not recurrent. The findings of that case report and the present cases suggest that MDS may be the underlying cause of NP. NP may be cured once MDS has been radically treated by bone marrow transplantation. Due to the lack of donors, the present cases were not treated by bone marrow transplantation.

In conclusion, if NP is diagnosed, a thorough examination for hematological disorders is mandatory. However, due to the rarity of such cases, the exact pathogenesis of NP associated with MDS requires further investigation.

## Figures and Tables

**Table I:** Classification of neutrophilic dermatoses.

Location of neutrophilic infiltrate	Type of neutrophilic dermatosis
Epidermis	Sneddon-Wilkinson disease
Dermis	Sweet’s syndrome
Dermis, vessels	Erythema elevatum diutinum
Dermis, eccrine glands	Neutrophilic eccrine hidradenitis
Dermis and hypodermis	Pyoderma gangrenosum
Hypodermis, fat lobules	Neutrophilic panniculitis
Hypodermis, septa	Sweet’s syndrome with subcutaneous involvement; erythema nodosum
Hypodermis and underlying tissues	Aseptic abscess

**Table II:** Clinical characteristics of patients with NP and MDS.

Characteristics	Case 1	Case 2
Age (years)	35	46
Gender	Male	Male
Main symptom	Recurrent high fever	Recurrent high fever
Other symptoms	Erythematous nodules over extremities and waist	Erythematous nodules over lower limbs and feet
Hepatomegaly and splenomegaly	Present	Absent
Hematological disorders	MDS and RCMD	MDS and RCMD
Treatment	Methylprednisolone (1 mg/kg)	Methylprednisolone (1 mg/kg)
Survival time (months)	16^a^	48^a^

a Until the present study.

NP, neutrophilic panniculitis; MDS, myelodysplastic syndrome; RCMD, refractory cytopenia with multilineage dysplasia.
